# The College Salaries Committee

**Published:** 1919-04-12

**Authors:** E. Grant

**Affiliations:** Secretary, Salaries Committee. 7 Henrietta Street, Cavendish Square, W. 1


### THE EDITOR'S LETTER-BOX.

**[Correspondence on all subjects is invited, but we cannot in any way be responsible for the opinions expressed by our correspondents, who must give their name and address as a guarantee of good faith, but not necessarily for publication. Correspondents are reminded that brevity of style and conciseness of statement greatly facilitate early insertion.]**

### The College Salaries Committee.

*To the Editor of* The Hospital.

Sir,—May I through the medium of your paper, on behalf of the Salaries Committee appointed by the Council of the College of Nursing, thank the matrons and secretaries of nursing institutions, the medical officers of health and the nurses, who have so readily given information regarding the salaries and the conditions of employment of nurses?

It is impossible at present to answer all the letters separately, but the Committee hopes that after the report is issued to the Council of the College that this may be done. Meantime the Salaries Committee will be glad if the writers will accept this assurance that their letters are receiving every consideration.—I am, Sir, yours faithfully,

E. Grant, Secretary, Salaries Committee.

7 Henrietta Street, Cavendish Square, W. 1,
April 4, 1919.

